# Fragmentation, dehumanization, commodification: crisis of medicine

**DOI:** 10.3325/cmj.2023.64.208

**Published:** 2023-06

**Authors:** Aleksandar Džakula, Dorja Vočanec, Karmen Lončarek

**Affiliations:** 1 Department of Social Medicine and Organization of Health Care, Andrija Štampar School of Public Health, University of Zagreb School of Medicine, Zagreb, Croatia; 2 Center for Integrated and Palliative Care, University of Rijeka, Faculty of Medicine, Rijeka, Croatia

Medicine is all the practices used by a certain population in a certain time and space to preserve health.

José María López Piñero

Most of the health successes in the last 150 years, in particular the development of sanitation and housing, have been achieved through political means, based on the biopsychosocial model of health (1). Mortality and morbidity from infectious diseases have been reduced to historically low levels. What remains to be dealt with, non-communicable diseases, is left to medicine as a narrow profession. There is even talk of medicine as a paradigmatic case of a radical monopoly (2). Essentially, the emergence of the phrase “medical delivery systems dry up the non-therapeutic sources of health” can be attributed to the practical implication that investments in medical technologies have led to a crisis in basic non-medical forms of care (3). For example, the development of expensive diagnostic devices or immunological drugs has diverted attention from the need for the development of comprehensive geriatric and palliative care.

Almost no one questions the idea that technological development is crucial for the further development of health care. We are bombarded daily with news about emerging health technologies, surgical procedures, and medicines. At the same time, we are bombarded with news about rising costs and a lack of resources in health care. Technology, of course, costs a lot, and requires highly educated professionals.

The way technology development is financed supports the fragmentation of care. Likewise, in many health care systems, financial incentives are structured in a way that encourages volume-based care rather than patient-centered, coordinated care. This can lead to a focus on specific procedures or services rather than on comprehensive and integrated care. Integration and coordination are too abstract for simple economic analyses or direct financial incentives (4,5). The direct beneficiaries of integration and coordination are not patients, but professionals, while patients as beneficiaries receive better care only when a certain threshold of coordination in the provision of medical services is achieved. Thus, the biomedical model of health has far supplanted the bio-psycho-social model, even though 80%-90% of variable health outcomes depend on the social determinants of health (6).

The modern system of education of health professionals follows the biomedical model and reinforces fragmentation through the development of specializations and subspecializations. The primary goal of this system is to create a professional who will serve the system well (7). This is why patients are often (or mostly!) given the least priority in the education system.

It is a truism that an organized system cannot do without professionals. However, the question is rarely asked whether professionals can do without an organized system, especially when it comes to the medical profession. Once upon a time, a doctor used to visit patients as a packman with his doctor's bag. Today's doctors need a rich health care ecosystem to truly be what they were trained for, and for their individual competencies to find application and purpose (8). In contrast to health care, social care is not so technologically advanced. The main tools of social care are still counseling, financial support, and the provision of existential, technologically extremely simple minimums, such as food, clothing, and housing.

A divergent development of health and social care is both a cause and a consequence of the biomedical model of health. However, a person's complete needs correspond to the biopsychosocial model of health (9). The fragmentation of comprehensive care into social care and health care, as well as the fragmentation of health care and the formation of interest subsystems, raise the question: do patients become redundant in such a system? If the system is not built around those who fight for their lives, then health care is no longer care for people but a commodity, professionals are service providers, and patients are consumers.

## The urge for coordination and integration

In the shadow of the resource crisis and the enthusiasm for innovation, we carried out the transition of medicine toward dehumanization and commodification. Who is the biggest loser here? In social and systemic crises, as a rule, losers are all those who are deprived of resources, either economically or socially.

However, the COVID-19 pandemic highlighted the problem of coordination of care in all segments of care and for all patient populations (10,11). Needs exist, knowledge and information exist, resources exist, but this is not enough. This failure is attributed to the specificity of the patient and the complexity of his or her condition. In other words, there are many different problems that should be solved in a coordinated manner, but coordination has not become a priority. Complex problems in health care are nothing new, nor are the specifics of each individual patient. So, why has the problem of complex patients suddenly become so dramatic?

The development of medical technologies has enabled us to successfully treat diseases and live long lives even with the most difficult diagnoses. The development of digital technologies, as well as transportation, has made innovations available and accessible. On the other hand, the demographic transition of society changed relations in primary social communities, and resource limitations have caused many people to face dramatic combinations of various social-medical problems. Health, social, mental, and other problems faced by the patient at the same time are covered by the term “complex patients.” A challenge for everyone. A big challenge with extremely negative trends.

Up to five percent of the population are considered complex patients. Caring for them sometimes requires half of all the health care resources (12,13). But an even greater problem are the resources that we need but do not have or do not use.

## Frustrated doctor, suffering patient

The frustrations of professionals arise from the crisis of the medical profession, because the foundations on which it rested for decades - the monopoly of knowledge, autonomy, and self-regulation - have been shaken (14). The development of society, and especially the emergence of digital technologies (“doctor Google,” AI...), have strongly changed the asymmetry of knowledge, and thus the roles of power and the status of doctors in society. The speed of the changes did not allow adequate transformation, so modern medicine is firmly trapped by many unrealistic expectations and unused opportunities.

In addition, the framework of medical diagnosis is only one of several lenses through which we view a person's individual situation and experience. No matter how much it is perfected, it cannot fully describe and respond to illness or health (15). It follows that doctors do not have exclusivity in illness and health, a state of affairs that represents a change in the decades-old paradigm.

Never in history have doctors been able to provide more therapies to help their patients. Never in history have doctors had such powerful tools and working conditions, but never have we witnessed so many people who cannot use the help we can offer them. The paradox of modern health care is summed up in the suffering of complex patients.
